# Evaluation of an educational intervention to increase HIV-testing in high HIV prevalence general practices: a pilot feasibility stepped-wedged randomised controlled trial

**DOI:** 10.1186/s12875-018-0880-9

**Published:** 2018-12-13

**Authors:** Charlotte F. Davies, Joanna M. Kesten, Mark Gompels, Jeremy Horwood, Megan Crofts, Annette Billing, Charlotte Chick, Margaret T. May

**Affiliations:** 1 Bristol Medical School, University of Bristol, Canynge Hall, 39 Whatley Road, Bristol, BS8 2PS UK; 2 Bristol Medical School, University of Bristol, Oakfield House, Oakfield Grove, Bristol, BS8 2BN UK; 3 National Institute for Health Research Collaborations for Leadership in Applied Health Research and Care West (NIHR CLAHRC West), University Hospitals Bristol, NHS Foundation Trust, 9th Floor, Whitefriars, Lewins Mead, Bristol, BS1 2NT England; 4 National Institute of Health Research (NIHR) Health Protection Research Unit (HPRU) in Evaluation of Interventions, Bristol Medical School, University of Bristol, Oakfield House, Oakfield Grove, Bristol, BS8 2BN UK; 5 Department of Immunology, Southmead Hospital, North Bristol NHS Trust, Westbury-on-Trym, Bristol, BS10 5NB UK; 6 Genitourinary medicine, Unity Sexual Health, Bristol Sexual Health Services, Tower Hill, Bristol, BS2 0JD UK; 7 NHS Bristol, North Somerset and South Gloucestershire CCG, South Plaza, Marlborough Street, Bristol, BS1 3NX UK

**Keywords:** General practice, HIV testing, Primary care, Step-wedged RCT, Education intervention, Feasibility study, Implementation study

## Abstract

**Background:**

HIV-infected patients often present to primary care several times with HIV-indicator conditions before diagnosis but the opportunity to test by healthcare professionals (HCPs) is frequently missed. Current HIV testing rates in primary care are low and educational interventions to facilitate HCPs to increase testing and awareness of HIV are needed.

**Method:**

We implemented a pilot feasibility stepped-wedged randomised controlled trial of an educational intervention in high HIV prevalence practices in Bristol. The training delivered to HCPs including General Practitioners (GP) aimed to increase HIV testing and included why, who, and how to test. The intervention was adapted from the Medical Foundation for HIV and Sexual Health HIV Testing in Practice (MEDFASH) educational tool. Questionnaires assessed HCP feedback and perceived impacts of the intervention. HIV testing rates were compared between control and intervention practices using 12 monthly laboratory totals.

**Results:**

169 HCPs (from 19 practices) received the educational intervention. 127 (75%) questionnaires were completed. Delivery of the intervention was received positively and was perceived as valuable for increasing awareness, confidence and consideration of testing, with HCPs gaining more awareness of HIV testing guidelines**.** The main pre-training HIV testing barrier reported by GPs was the patient not considering themselves at risk, whilst for nurses it was a concern about embarrassing or offending the patient. Most HCPs reported the intervention addressed these barriers. The HIV testing rate increased more in the control than in the intervention practices: mean difference 2.6 (95% CI 0.5,4.7) compared with 1.9 (− 0.5,4.3) per 1000 patients, respectively. The number of HIV tests across all practices increased from 1154 in the first 6 months to 1299 in the second 6 months, an annual increase in testing rate of 2.0 (0.7,3.4) from 16.3 to 18.3 per 1000 patients.

**Conclusion:**

There was a small increase in HIV testing rates over the study period, but this could not be attributed to the educational intervention. More effective and sustainable programmes tailored to each practice context are needed to change testing culture and HCP behaviour. Repeated training, supported by additional measures, such as testing prompts, may be needed to influence primary care HIV testing.

**Electronic supplementary material:**

The online version of this article (10.1186/s12875-018-0880-9) contains supplementary material, which is available to authorized users.

## Background

Approximately 101,200 people in the United Kingdom (UK) are living with human immunodeficiency virus (HIV), of these 13% are undiagnosed and unaware of their infection [1]. The majority of HIV transmissions are from untreated (often undiagnosed) individuals. Patients are unlikely to transmit HIV to sexual partners if they are diagnosed and treated with effective anti-retroviral therapy (ART) [2]. In 2015, 39% of newly diagnosed adults were diagnosed late in England [3, 4]. Late diagnosis of HIV is associated with increased hospitalisation, decreased life expectancy [5–7] and higher treatment and care costs [8].

Recent audits and reviews show a clear need to increase and improve HIV testing in the UK particularly within primary care [9, 10]. Routine testing in general practice in the UK is recommended in areas with > 2 per 1000 population diagnosed prevalence [11] based on cost-effectiveness studies of HIV testing in the USA and France [12–14]. Healthcare professionals (HCPs) in primary care have opportunities to initiate HIV testing in at risk individuals and thereby reduce the proportion of undiagnosed infection in the UK [15, 16]. Patients with HIV often see their General Practitioner (GP) with an HIV indicator condition (IC) (a sign, symptom or diagnosis that is more commonly found in those with HIV) several times before their HIV is detected [15–22]. Due to the stigma surrounding HIV there are often barriers to testing experienced by both the HCP and the patients. Literature reviews reveal that the barriers HCPs experience barriers to HIV testing including lack of confidence or anxiety around offering a test, concern about offending or upsetting patients [23], privacy and confidentiality issues [23] and insufficient knowledge or training [24, 25]. There are also structural and organizational barriers such as lack of time during consultations and limited resources allocated to HIV testing [26]. A more proactive offer of a test by the HCP could increase HIV testing rates [25] and thereby reduce undiagnosed infection and late diagnosis in the UK, as recommended by national HIV testing guidelines [18, 27]. Routine testing of HIV could also help reduce the stigma attached to HIV [28].

Interventions to address these issues in primary care include expanding HIV testing by screening all newly registered patients [29–31]. Opt-out testing in 8 pilot studies (including 2 within primary care) were shown to be feasible, acceptable and cost-effective [32]. A recent cluster randomised controlled trial (RCT) in Hackney (London UK) offered opt-out rapid point of care testing (POCT) alongside education sessions to newly registered adults in comparison to usual care, resulting in increased rates of HIV diagnosis [31]. The study was shown to be cost-effective in the medium term in settings with extremely high HIV incidence, defined as > 5 /1000 population [33]. Educational interventions for GPs and nurses within primary care aimed at increasing HIV testing rates have also shown encouraging results [34–36]. However, most of these studies have taken place in extremely high HIV prevalence cities in the UK such as London and Brighton. Therefore, it is not known whether similar results will be obtained in high diagnosed prevalence practices (defined as > 2 per 1000) in a city with estimated HIV prevalence similar to the national average for England, currently 2.3/1000 population aged 15–59 years [37].

This implementation study aimed to investigate the feasibility of a stepped-wedge RCT to evaluate an educational intervention’s appropriateness, usefulness and effectiveness on increasing HCPs HIV testing rates in high HIV diagnosed prevalence practices in Bristol, a city with an overall estimated HIV prevalence of 2.5/1000 population aged 15–59 years [37]. Qualitative interviews with HCPs were also undertaken at least 3 months post-intervention to explore how the training was experienced by HCPs and to understand the perceived impacts of the intervention on HIV testing and explore the barriers to testing in more detail. These results are reported elsewhere [38].

## Methods

### Design of pilot feasibility stepped-wedged RCT

**Fig. 1:**
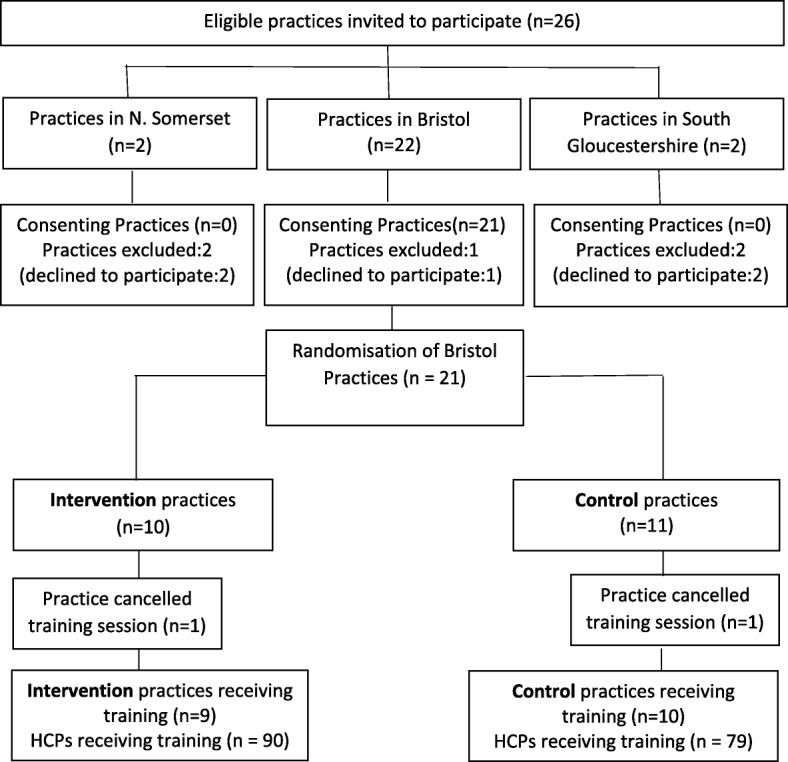
Practice recruitment into the stepped wedged RCT of the HIV testing education intervention

This was a pilot feasibility stepped-wedge RCT which invited 26 GP practices in Bristol, South Gloucestershire and North Somerset with high practice population HIV diagnosed prevalence (> 2 per 1000) to take part. Practices that agreed to take part were randomised to either the intervention arm (first to receive training) or to the control arm (second group to receive training) using a Stata random allocation program (Fig. 1). Training sessions were delivered between October 2015 and March 2016 in the intervention arm and from April to July 2016 in the control arm. The intervention was delivered at a rate of approximately two practices per month over roughly 10 months. The first two practices acted as pilots which informed tailoring of the training before further roll out.

### Details of intervention

We chose the format of the intervention, a one-hour interactive workshop delivered within general practices by a genito-urinary medicine (GUM) specialist registrar, in consultation with GPs who indicated that due to workload pressures they would only be able to commit to attend to a single short session. All staff at the practice were invited to attend, not just clinical staff with a particular interest in sexual health. The content of the training was designed to increase testing and was based on the Medical Foundation for HIV and Sexual Health (MEDFASH) HIV Testing in Practice (TIPs) educational tool (http://www.medfash.org.uk/welcome-to-hiv-tips) which was adapted for local use, for example, by using locally relevant statistics and signposting to local services. TIPS was already a nationally-approved training programme and therefore we thought that, if successful, it could be easily rolled out in other areas. The training provided a short knowledge assessment quiz, an update on current BHIVA and NICE HIV testing recommendations and HIV associated clinical ICs and covered barriers to HIV testing that the practice team had or may have encountered and explored ways to overcome these. The presentation included a summary of all the ICs, but highlighted those that occur more commonly in primary care, such as seroconversion illness, rash, oral and gut conditions, dermatology, respiratory and other “non-specific” weight loss/sweats/abnormal blood count presentations. Conditions were grouped “syndromically” to reduce confusion and increase memorability. The training also included the following topics: why test and the importance of testing, who to test, how to do a test including the discussion with the patient, how to handle both negative and positive results and linkage to care. The training concluded with a discussion about how the practice could increase testing, which included case study examples. After the training, each practice received a summary sheet on the information covered which included references to useful resources (including the MEDFASH TIPs website link).

### Analysis

#### Quantitative evaluation of HIV testing

**Fig. 2:**
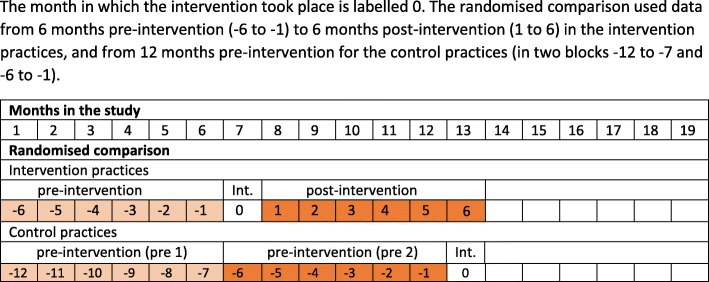
Timeline

Numbers of HIV tests at the practice level and their results were obtained from the Public Health England (PHE) South West laboratory at Southmead Hospital. These were aggregated monthly by practice. Routine tests done by midwives for antenatal screening were excluded. Data on practice population size were obtained from the primary care commissioners and used to convert frequencies to annualised rates per 1000 patients. The month of the intervention was coded as zero, with number of months pre-intervention and post-intervention labelled negative and positive, respectively (Fig. 2).

We calculated the difference in HIV testing rates by practice and overall in 6-month blocks. For the randomised comparison we compared the differences in pre- and post-intervention rates in the intervention practices with the differences in rates in the matched time periods in control practices (all prior to intervention) and tested for a difference in differences between the arms to account for variation between practices. We estimated the positivity rate for the HIV tests in the intervention and control practices combined.

#### Questionnaire evaluation of education intervention

A questionnaire evaluation was also undertaken immediately after the training by participants to obtain feedback on the appropriateness and usefulness of the intervention. The questionnaire format involved scoring statements and providing open text responses. An example questionnaire is shown in the Additional file 1. Mean scores for seven statements were calculated using a 4-point Likert scale (1 = strongly disagree, 2 = disagree, 3 = agree, 4 = strongly agree).

## Results

Of the 26 General Practices invited to participate, 21 agreed to take part and were randomized (10 to intervention and 11 to control arms). Of note, none of the four practices outside Bristol agreed to participate. One practice in each arm did not receive training because of logistical issues (no convenient time available) and therefore training sessions were delivered to 9 practices in the intervention arm and later to 10 practices in the control arm (Fig. 1).

### Quantitative evaluation of HIV testing

Practice HIV testing rates were compared pre-and post-intervention and between control and intervention practices using a randomised comparison. Figure 2 shows the timeline for the randomised comparison. In the intervention practices, the number of HIV tests in the 6 months pre-intervention was 574 which increased to 631 in the 6 months post intervention, corresponding to an increase in annual HIV testing rate from 17.4 to 19.1 per 1000 patients (Table 1). The number of HIV tests in the control arm also increased from 580 to 668 in the same time period, corresponding to an increase in annual HIV testing rate from 15.3 to 17.6 per 1000 patients. Over all the practices the increase in annual HIV testing rate per 1000 patients was 2.0 [95% Confidence Interval (CI) 0.7, 3.4] and was similar in the intervention [1.7 (− 0.3, 3.8)] and the control [2.3 (0.5, 4.1)] practices. The mean difference in testing rates was 1.9 (− 0.5, 4.3) and 2.6 (0.5, 4.7) across the intervention and control practices, respectively, a difference of differences of − 0.7 (*p* = 0.68) in favour of the control arm which could have been due to chance. Any increase in testing was likely due to secular trend rather than to the education intervention. In the intervention arm, there were 6 positive tests, 3 pre- and 3 post-intervention. In the control arm there were 9 positive tests, 7 in the first and 2 in the second period. During the 12 months of the study there were a total of 2453 HIV tests recorded of which 15 were positive, a positivity rate of 6.1 per 1000 tests which exceeds the 2 per 1000 threshold for prevalence of undiagnosed HIV considered to be cost effective in primary care HIV screening [39].

**Table 1:** Randomised comparison between intervention and control practices. Number of HIV tests, annual rate of HIV testing per 1000 patients, and difference in rate by practice and randomisation arm and overall, and number of positive tests. Data for 6 months pre and post intervention for intervention practices, and for corresponding time periods (both pre-intervention) for the control practices

		Randomised comparison				
	Total population	No. of HIV tests		Annual rate of HIV tests per 1000 patients		
		Pre	Post	Pre	Post	Difference
Intervention practices		−6,-1	1,6	−6,-1	1,6	post-pre
1	5547	99	90	35.7	32.4	−3.2 (−13.0, 6.5)
2	6295	112	122	35.6	38.8	3.2 (−6.3, 12.7)
3	12,794	103	105	16.1	16.4	0.3 (−4.1, 4.7)
4	8506	52	44	12.2	10.3	−1.9 (−6.4, 2.6)
5	7206	40	47	11.1	13.0	1.9 (−3.1, 7.0)
6	7203	48	57	13.3	15.8	2.5 (−3.1, 8.1)
7	6304	60	67	19.0	21.3	2.2 (−4.8, 9.2)
8	5496	15	21	5.5	7.6	2.2 (−2.1, 6.5)
9	6710	45	78	13.4	23.2	9.8 (3.4, 16.3)
Mean difference *n* = 9						1.9 (−0.5, 4.3)
TOTAL intervention	66,061	574	631	17.4	19.1	1.7 (− 0.3, 3.8)
Positive tests		3	3			
		Pre 1	Pre 2	Pre 1	Pre 2	Difference
Control practices		−12,-7	−6, −1	−12,-7	−6, −1	Pre2-Pre1
10	9081	36	36	7.9	7.9	0.0 (−3.7, 3.7)
11	4133	16	22	7.7	10.6	2.9 (−2.9, 8.8)
12	10,833	63	76	11.6	14.0	2.4 (−1.9, 6.7)
13	14,547	149	159	20.5	21.9	1.4 (−3.4, 6.1)
14	2878	38	41	26.4	28.5	2.1 (−10.0, 14.2)
15	4711	18	27	7.6	11.5	3.8 (−1.8, 9.4)
16	12,456	108	115	17.3	18.5	1.1 (−3.6, 5.8)
17	6945	92	132	26.5	38.0	11.5 (3.1, 20.0)
18	3334	16	18	9.6	10.8	1.2 (−5.7, 8.1)
19	6960	44	42	12.6	12.1	−0.6 (−5.8, 4.6)
Mean difference *n* = 10						2.6 (0.5, 4.7)
TOTAL control	75,878	580	668	15.3	17.6	2.3 (0.5, 4.1)
Positive tests		7	2			
All practices						
Mean difference *n* = 19						2.3(0.7, 3.8)
TOTAL all (pooled)	141,939	1154	1299	16.3	18.3	2.0 (0.7, 3.4)
TOTAL positive tests		10	5			

### Questionnaire evaluation of education intervention: HCPs feedback on training

In total, 169 HCPs (93 GPs, 53 nurses and 23 `others’) received the training. The ‘other’ group consisted of healthcare assistants (HCA), practice managers, assistant practice managers and medical students. On average 9.3 HCPs per practice received the training (range 4 to 17). Feedback was available from 127 evaluation questionnaires out of a possible 169 (75% response rate) which participants completed immediately after the training. Some HCPs left the session early and were too busy to complete the questionnaire. Questionnaire participant characteristics are shown in Table 2.

**Table 2:** Evaluation Questionnaire and interview participant characteristics

General Practice Number	Evaluation Method	GP (*n*)	Nurses & Other^a^(*n)*	Gender of HCP interviewed Male/Female	Questionnaires Total (*n*)
1	Interview Questionnaire	1 4	1 6	1/1	10
2	Interview Questionnaire	0 8	1 4	0/1	12
3	Interview Questionnaire	0 2	1 2	0/1	4
4	Interview Questionnaire	2 4	2 2	0/4	6
5	Interview Questionnaire	0 3	2 6	1/1	9
6	Interview Questionnaire	2 4	0 1^b^	1/1	5
7	Interview Questionnaire	1 4	0 2	0/1	6
8	Interview Questionnaire	0 3	1 6	0/1	9
9	Interview Questionnaire	6 7	1 3	2/5	10
10	Interview Questionnaire	0 4	1 2	0/1	6
11	Interview Questionnaire	1 4	0 2	0/1	6
12	Interview Questionnaire	2 6	0 1	0/2	7
13	Interview Questionnaire	1 3	0 4	0/1	7
14	Interview Questionnaire	0 3	0 2	0/0	5
15	Interview Questionnaire	0 7	0 1	0/0	8
16	Interview Questionnaire	0 2	0 1	0/0	3
17	Interview Questionnaire	0 2	0 2	0/0	4
18	Interview Questionnaire	0 4	0 2	0/0	6
19	Interview Questionnaire	0 3	0 1	0/0	4
Overall totals					127

(^a^Advanced Nurse Practitioner and Healthcare Assistants, ^b^Questionnaire completed by a Clinical Pharmacist, HCP: Healthcare Professional. Practices 14 to 19: no interviews took place at these practices, only evaluation questionnaires were completed)

**Fig. 3:**
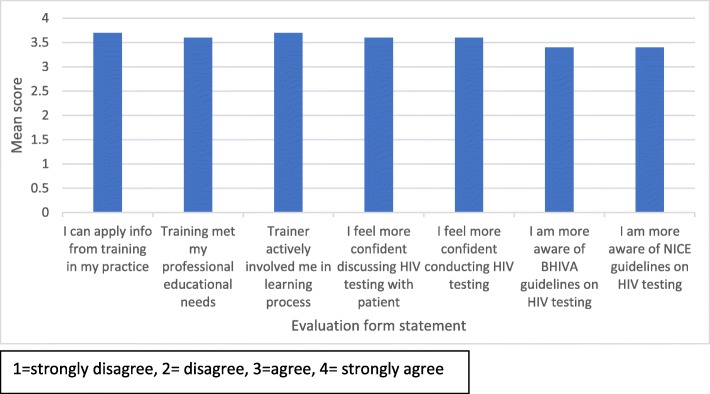
Results of evaluation questionnaire completed by healthcare professionals attending the intervention

All mean scores were above 3 (‘agree’) for the statements shown in Fig. 3. The following statements ‘*I can apply the information gained from the training in my practice setting* ‘ and ‘*The trainer actively involved me in the learning process*’ scored the highest mean level of agreement (both statements scoring 3.7). The following statements ‘*The training met my professional educational needs*’ and ‘*As a result of the training I feel more confident in my ability to discuss HIV testing with a patient’* and ‘*more confident in my ability to conduct HIV testing*’ had a mean score of 3.6. Statements that attendees were ‘*more aware of the BHIVA and NICE guidelines on HIV testing*’ scored the lowest mean level of agreement (both scored 3.4).

The four most frequently cited barriers to HIV testing reported in the questionnaire by GPs prior to training were: 1) “*The patient doesn’t consider themselves at risk*” 2) “*Concern about embarrassing or offending the patient*” 3) “*I don’t want to scare my patient when their symptoms probably aren’t HIV related*” and 4) “*There isn’t time to discuss HIV*”.

The four most frequently cited barriers reported by nurses were 1) “*Concern about embarrassing or offending the patient*” 2) “*The patient doesn’t consider themselves at risk*” 3) “*I don’t know how to manage HIV*” and 4) “*What would I do if I found an HIV positive*” and “*I don’t want to scare my patient when their symptoms probably aren’t HIV related”.*

When GPs and nurses were asked *“Did the training session adequately address the barriers that apply to you and/or your practice and how to overcome these?”* the majority of GPs (94%) and practice nurses (78%) replied that the intervention had adequately covered the barriers as well as ways/techniques to overcome them*.*

Questionnaire data showed that delivery of the HIV training was received positively by the majority of HCPs, it was perceived as valuable for increasing awareness of HIV ICs, confidence and consideration of testing and HCPs gained more awareness of BHIVA and NICE HIV testing guidelines. HCPs scored highly for feeling more confident around discussing and conducting an HIV test immediately post training.

## Discussion

This study has shown that although an HIV testing educational intervention was received very positively by HCPs this did not result in increased HIV tests. We showed that HIV testing rates did increase over the study period, but this was a secular trend rather than due to the intervention and occurred in both control and intervention arms of the trial. Possible explanations for the lack of effect of the education intervention on testing rates include weak design features such as one-off training compared to more sustainable programmes with regular reinforcement training and performance feedback, the focus on information rather than skills-based training which may have failed to overcome communication barriers experienced by HCPs in offering HIV tests, and that chosen practices may have already reached saturation point for HIV testing because they were those with the highest diagnosed prevalence of HIV in Bristol. Organisational and structural barriers included severely limited resources available for delivering this intervention due to local authority budget restrictions. Furthermore, GP practices were very limited in their available time to dedicate to further training even if it had been affordable. There is increasing pressure on GPs to undertake more health surveillance with less resource which has probably contributed to GPs prioritising other areas above testing for HIV [26]. Inadequate consideration of the local context may have thwarted implementation of the desired behaviour change needed to increase HIV testing in general practice, even when evidence of the benefit of increased testing in reducing late diagnosis was clearly presented [40].

### Strengths and limitations

A stepped-wedged RCT is a novel way to evaluate the effects of an educational intervention. It incorporates a fair process of determining the order of intervention rollout where there are logistical constraints, as it cannot be delivered to all practices simultaneously. The RCT approach also has advantages over audit as it minimises selection bias as to which practices received the intervention and which were controls. We compared testing rates in a randomised comparison that accounted for trends in testing over time. As this was a pilot feasibility study, the number of general practices was small, and the data collection time period was relatively short. In particular, there were too few positive tests recorded to inform whether the education intervention improved targeting or appropriateness of testing. Furthermore, tests were anonymised therefore we could not determine for those who tested positive whether they had a prior HIV diagnosis or whether they linked to care.

We targeted delivery of the training to practices with a high diagnosed HIV prevalence (2–5 per 1000 population) in the practice population which may mean that these findings are not generalisable to practices with different diagnosed HIV prevalence rates [41]. The practices in our study had very high testing rates before the intervention (16.3 per 1000 population), which were much higher than the national average for general practice (10.1 per 100 and 4.4 per 1000 population in very high and high prevalence areas, respectively). Therefore, the capacity to increase testing rates in participating practices may have been smaller than in practices not in the study that test less. In East London where there have been interventions to increase HIV testing [39], there were pre-existing local sexual health enhanced services, but this was not the case in Bristol. However, the practices with higher diagnosed prevalence in Bristol would have had an increased awareness of testing due to having registered HIV positive patients which may explain their already high testing rates.

The testing data is at the practice level and therefore it was not possible to examine variation in testing at the HCP level. The effects of the intervention may have been diluted if only a small proportion of HCPs in the practice attended the training. A one-off session may have limited attendance rates compared to training sessions given frequently, which would have allowed more HCPs the opportunity to attend. However, a one-off session reduces the burden on HCPs which may have encouraged more to attend.

In the UK, the population of pregnant women is very low risk for HIV, with testing rates around 96%. Therefore, this population is different from those who are offered an HIV test during usual primary care consultations. We excluded HIV tests that were known to be carried out by midwives, but some tests that were carried out as part of ante-natal screening may have inadvertently been included. This would tend to raise testing rates, but result in fewer positive tests, but this should not have been differential between the arms of the trial. We considered positive HIV tests to be newly diagnosed patients, but it is possible that the result could have been from a repeat test after a positive test during ante-natal screening, or a patient with diagnosed HIV that was transferring from another practice or had not declared their HIV status to the GP.

### Our study in context

Other sexual health testing educational interventions have been undertaken in the UK [35, 42]. HIV testing rates increased in general practices receiving a multifaceted educational intervention (Sexual Health in Practice -SHIP) in an extremely high diagnosed HIV prevalence area in London [35] and that these were sustained over 8 years [43]. This study used five rounds of training over a 24-month period which differs to the one-off training used in the current study, potentially illustrating the benefits of repeated training sessions over time. Other differences included a broader focus on sexual health, separate peer led training for GPs and practice nurses, and a focus on practicing skills, particularly rapid sexual risk assessment and verbal strategies to overcome barriers to testing.

A national pilot of an educational intervention known as 3Cs and HIV study was undertaken by PHE to improve general practice staff skills and confidence to increase chlamydia testing rates, provide condoms with contraceptive information and carry out HIV testing according to national guidelines [42]. Similarly, to our study, results of this pilot showed that the short educational sessions had no impact on chlamydia testing and that there remained barriers preventing testing intentions being translated into measurable changes in test numbers [42]. There were also many barriers to testing reported by GPs and nurses in our study which need to be actively addressed to reduce undiagnosed infection. Similar barriers were reported from qualitative interviews which took place after the HIV testing workshops in the 3Cs and HIV study which revealed that staff still lacked confidence and experience of offering HIV tests in routine consultations [44].

As part of our study a qualitative researcher carried out interviews with HCPs who attended the training approximately 3-months post-training. The HIV training was experienced positively and improved perceived awareness, confidence, and consideration of HIV testing whilst perceptions of testing rates were mixed. Continued barriers to testing included perceived lack of opportunity to consider HIV during consultations. The study, which is reported more fully elsewhere [38], concluded that repetition may be needed to sustain the impact of the education intervention.

### Implications for research and/or practice

Increasing the uptake of HIV testing across healthcare settings [3] and reducing the stigma surrounding HIV testing is still a major priority in the UK because of late HIV diagnosis with advanced disease [15, 45, 46]. However, we cannot tell from our data whether the rate of HIV testing in our study practices was sufficiently high already to meet the requirements of guideline testing. For the level of HIV testing to reach saturation in areas of high diagnosed HIV prevalence, all new patients registering with a GP, those having a blood test who have not tested for HIV in the previous 12 months, those from high risk groups, those who have travelled to endemic regions, and those with HIV indicator conditions should be offered an HIV test in accordance with BHIVA and NICE recommendations [3]. Opt-out testing can facilitate increased testing as has been evident with the successful national policy recommendation introduced in 1999 that all pregnant women should have an HIV test alongside other antenatal screening tests (via an opt-out approach). The policy has a 96% acceptance rate in antenatal settings and has had a dramatic effect on reducing the number of women with undiagnosed HIV post-delivery and mother-to-child transmission [32]. Studies promoting IC-based testing [43] have shown a relatively low increase in testing compared to HIV screening studies such as the RHIVA trial of rapid HIV testing [31], although positivity rates tend to be higher in targeted interventions [10].

The findings from this study have implications for policy-makers. Training on its own is not an effective behaviour change technique and future interventions to increase HIV testing should consider using behaviour change theory to develop a complex intervention. This might be based on the behaviour change wheel known as the “COM-B” system which considers capability (psychological and physical), opportunity (physical and social), and motivation (reflective and automatic) to change behaviour [47]. Single training sessions on HIV testing, although perceived positively by HCPs, are likely to require repetition and support from additional interventions or strategies to help encourage increased HIV testing rates. Computer prompts based on risk algorithms are one strategy to support HIV testing. Prompts would notify the HCP when patients show HIV ICs or behavioral risk factors (e.g. drug use, unprotected sex). Studies using clinical reminders or computer prompts based on HIV risk factors have shown the benefits of this type of intervention, impacting significantly on HIV testing rates [48–53]. A recent initiative in the UK assessed the feasibility of an electronic clinical decision support system, prompting HIV testing based on doctors and nurses selecting certain other tests (e.g. hepatitis serology). The system was found to be useable and acceptable by hospital doctors, GPs and nurses and there was a 6% increase in testing rates over the 3-month study period [54]. A recent literature review has provided evidence that HIV ICs have the potential to be used more effectively as triggers for earlier HIV testing [55].

## Conclusions

Interventions to improve general practice HIV testing rates remain a priority. A single educational training session did not increase HIV testing rates in 19 practices in Bristol with high diagnosed HIV prevalence, despite HCPs reporting that the training was useful and that they felt more confident to offer testing. Commissioners and Public Health Officials need to understand the pitfalls and risks of a one-off training intervention in general practice and consider implementation of more effective and sustainable programmes. Better designed complex interventions tailored to the context of each practice are required to change HIV testing culture and HCP behaviour. Further educational sessions would need to be supported by different strategies to help reinforce and facilitate any long-term impact on HIV testing rates.

## Additional file

Additional file 1: Questionnaire Evaluation. (DOCX 38 kb)

## Abbreviations

ART: Anti-retroviral therapy
BHIVA: British HIV Association
GP: General Practitioner
GUM: Genito-Urinary Medicine
HCAs: Health Care Assistants
HCP: Healthcare professional
HIV ICs: HIV Indicator conditions
HIV: Human immunodeficiency virus
MEDFASH: Medical Foundation for HIV and Sexual Health
MSM: Men who have sex with men
NHS: National Health Service
PHE: Public Health England
POCT: Point of care testing
RCT: Randomised controlled trial
