# Supplementary material for: Evaluation of an educational intervention to increase HIV-testing in high HIV prevalence general practices: a pilot feasibility stepped-wedged randomised controlled trial
Source: BMC Fam Pract. 2018 Dec 13;19:195. doi: 10.1186/s12875-018-0880-9 (PMC6292019; doi:10.1186/s12875-018-0880-9)
Supplement: Supplementary file 1 — Questionnaire Evaluation. (DOCX 38 kb) [file 12875_2018_880_MOESM1_ESM.docx]

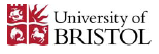
 [
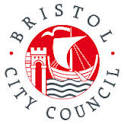
](http://www.google.co.uk/url?sa=i&rct=j&q=&esrc=s&frm=1&source=images&cd=&cad=rja&uact=8&ved=0CAMQjRxqFQoTCIC57YWuo8cCFQMDGgod-10IXA&url=http://www.first4adoption.org.uk/agency/bristol-city-council/&ei=ICPLVYCpEYOGaPu7oeAF&bvm=bv.99804247,d.d2s&psig=AFQjCNFsmDep5znoANXF8IAgCeozW4Bi2g&ust=1439462560321655)

Prof. Margaret May, School of Social & Community Medicine, University of Bristol, [m.t.may@bristol.ac.uk](mailto:m.t.may@bristol.ac.uk) 0117 9287287

Dr. Charlotte Davies, School of Social & Community Medicine, U. of Bristol, [Charlotte.Davies@bristol.ac.uk](mailto:Charlotte.Davies@bristol.ac.uk) 0117 9287209

**Improving HIV testing in Primary Care in Bristol**

**Training Evaluation Questionnaire**

**Job Title………………………….……………………………………………… Name of your Practice………………………………………………………………**

**No of Years practicing in Primary Care……………………………**

So that we can measure the success of this training, we would be very grateful if you would complete this short questionnaire based on the training you received today. It shouldn’t take any longer than about 10 minutes. Thank-you for your time.

1. **In the box below please tick by the number that best represents your knowledge and skills after the training**

| **Self-assessment of knowledge/skills related to :** | **Rating scale** | | |
| --- | --- | --- | --- |
|  | **1= Poor** | **2=Fair** | **3=Good** |
| Information on primary HIV infection |  |  |  |
| Information on HIV clinical indicator diseases |  |  |  |
| Overcoming practice barriers to HIV testing |  |  |  |
| Talking to patients about HIV testing |  |  |  |
| Referring patient on for HIV treatment /care if diagnosed HIV positive |  |  |  |

**Overall evaluation of training**

1. **Here is a list of the most commonly cited barriers and challenges that health professionals sometimes express, please tick against the statements that apply to you and/or your practice**

___ Concern about embarrassing of offending the patient

___ Maintaining patient privacy and confidentiality

___ I don’t know how to manage HIV

___ What would I do if I found a positive?

___ General practice is not funded to provide HIV testing

___ Concern over having to discuss patient’s sexual history

___ I am not confident about which conditions are associated with HIV

___ I don’t want to scare my patient when their symptoms probably aren’t HIV related

___ There isn’t time to discuss HIV

___ You need to be able to do special counselling to do an HIV test

___ The patient doesn’t consider themselves at risk

1. Did the training session adequately address the barriers that apply to you and/or your practice and how to overcome them? If not please state which barriers were not addressed

……………………………………………………………………………………………………………………………………………………………………………………………………………………………………………………………………………………………………………………………………………………………………………………

1. **Please answer the following questions, your comments will provide important feedback to us about the effectiveness of the training**
2. What do you feel were the strengths of the training?

………………………………………………………………………………………………………………………………………………………………………………………………………

………………………………………………………………………………………………………………………………………………………………………………………………………

1. Could there be any improvements made to the training? , if so please describe

……………………………………………………………………………………………………………………………………………………………………………………………………..

……………………………………………………………………………………………………………………………………………………………………………………………………..

1. Do you still have concerns around offering a patient an HIV test, if yes please explain

…………………………………………………………………………………………………………………………………………………………………………………………………….

…………………………………………………………………………………………………………………………………………………………………………………………………….

1. Is there anything else that would be useful to include in the training?

…………………………………………………………………………………………………………………………………………………………………………………………………….

…………………………………………………………………………………………………………………………………………………………………………………………………….

1. Will you do anything differently in your practice setting as a result of this training, if yes please explain

............................................................................................................................................................................................................

……………………………………………………………………………………………………………………………………………………………………………………………………..

1. **Please rate the following statements using agree and disagree categories below by ticking the appropriate boxes**

|  | **Rate** | | | |
| --- | --- | --- | --- | --- |
|  | **1= Strongly Disagree** | **2= Disagree** | **3 = Agree** | **4 = Strongly Agree** |
| I can apply the information gained from the training in my practice setting |  |  |  |  |
| The training met my professional educational needs |  |  |  |  |
| The trainer actively involved me in the learning process |  |  |  |  |
| As a result of the training I feel more confident in my ability to discuss HIV testing with a patient |  |  |  |  |
| As a result of this training, I am more confident in my ability to conduct HIV testing |  |  |  |  |
| I am more aware of the British HIV Association (BHIVA) National Guidelines on HIV testing |  |  |  |  |
| I am more aware of the National Institute of Clinical Excellence (NICE) Guidelines on HIV testing |  |  |  |  |

**Thankyou.**

**This questionnaire was developed by the University Of Bristol and supported by Bristol City Council**
